# Multiple metachronous malignancies, one patient with three primary malignancies: a case report

**DOI:** 10.1186/1752-1947-1-15

**Published:** 2007-05-02

**Authors:** Horace Fletcher, Gilian Wharfe, Elaine Williams, Barrie Hanchard, Derek Mitchell

**Affiliations:** 1 Department of Obstetrics and Gynaecology, University of the West Indies, Mona, Kingston, Jamaica; 2 Department of Pathology, University of the West Indies, Mona, Kingston, Jamaica; 3 Department of Surgery, University of the West Indies, Mona, Kingston, Jamaica

## Abstract

We present a 61 year old Para 4 woman who presented with stage II Infiltrating lobular carcinoma of the breast after modified radical mastectomy. She was treated with Tamoxifen for seven years. She was diagnosed with multiple myeloma during year seven post mastectomy because of wrist pain. She was treated with melphalan, prednisone and allopurinol which she tolerated well and the pain in the wrist improved. Tamoxifen was also stopped. Ten months later she presented with vaginal bleeding and was diagnosed with a poorly differentiated endometrial adenocarcinoma at hysteroscopic suction curettage and had an abdominal hysterectomy. Two years later the patient succumbed to metastatic endometrial cancer.

## Background

The development of a second primary cancer after treatment of the first with radiotherapy or chemotherapy is well documented [1]. This is often seen with hematological malignancies in childhood where other malignancies, usually haematologic follow, when there is good five year survival [1].

There are several other reasons for a patient to develop multiple primary malignancies. There may be a genetic predisposition resulting in the cancer family syndrome. The BRCA gene mutation would be one such example. They may also arise as a result of oncogenic viruses such as HPV and HTLV1. DNA damaging toxins are another cause for the development of these malignancies. Exposure to carcinogens can affect different organs at the same time, for example smoking can affect the lungs, nasopharynx and bladder while HPV affects the vulva, vagina and cervix. In some cases they are related to decreased tumour suppression in immunocompromised patients. They may also arise by chance as successful treatment of one malignancy causes prolongation of survival with the possibility of a second one occurring. We present a case of a woman who presented with three primary malignancies over a seven year period.

## Case presentation

**Figure 1:**
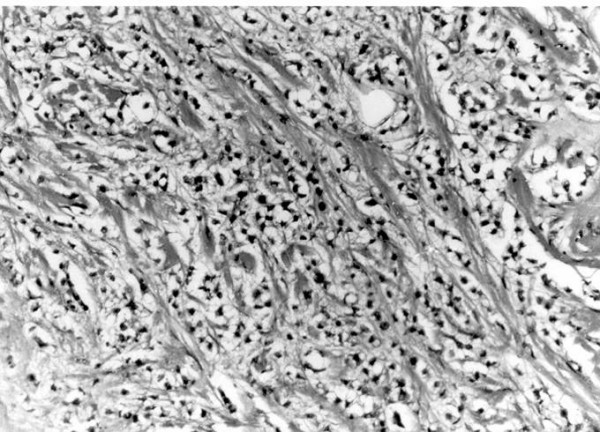


MW a 61 year old Para 4 presented in 1994, with a history of a lump in the breast for 5 years. She had noted an increase in size just prior to presentation. There was no associated nipple discharge, or pain and she gave no family history of breast cancer. In her past history she had been treated for glaucoma with pilocarpine and timolol maleate and had had surgical treatments for a tubal ectopic gestation and also for carpal tunnel syndrome. On general examination she was a middle aged woman in good health. There were no abnormal findings in her respiratory, cardiovascular, neurological or musculoskeletal systems. No masses were palpable in her abdomen and pelvic examination was normal. The left breast contained a 3 cm diameter firm mobile lump in the axillary tail which was not attached to the skin or the chest wall. The right breast was normal. Both fine needle aspiration and trucut biopsies of the lump proved to be inadequate for diagnosis but during the procedures it was noted that the lump was partially fixed, suggesting malignancy. Excision biopsy of the lesion proved on histology to be an infiltrating lobular carcinoma of the breast (figure 1). The patient elected to have a modified radical mastectomy. This specimen contained six (6) lymph nodes from the axillary dissection, all of which were free of metastasis. The deep resection margin was also free of tumour and she was designated stage II disease. No radiotherapy was given and although estrogen receptor studies were unavailable at that time, she was started on Tamoxifen.

**Figure 2:**
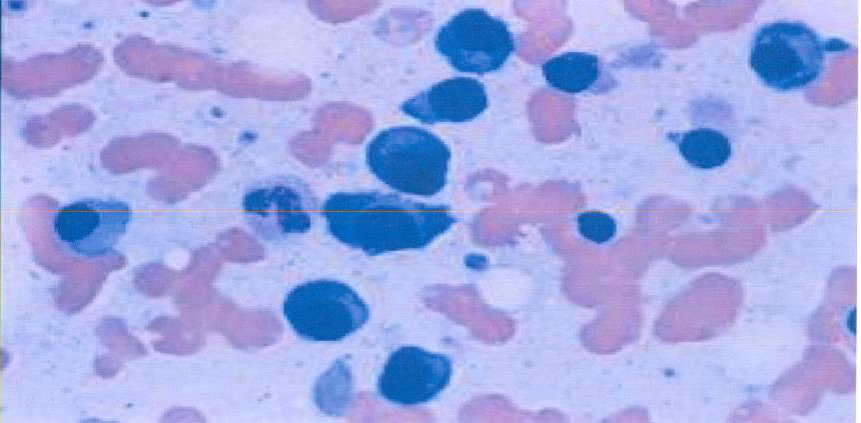


Five years later (1999) she presented with back pain. Radiographs of the spine revealed sclerosis in the body of the fifth lumbar vertebra. It was thought that this was possibly metastatic disease but a bone scan was reported as normal. Mammograms done every year on the remaining breast were also described as normal. A year later (2000), about 6 years post-mastectomy, there was no evidence of recurrence but she was continued on Tamoxifen. In February 2001, seven years after her initial presentation she presented with pain in the volar aspect of her right wrist for 2–3 months with restriction of movement. She was seen by the orthopaedic surgeons who found mild swelling with pain on radial deviation but no pain on compression, no anteroposterior laxity and no wasting. Radiography of the wrist showed a lucent area in distal ulna. This was confirmed by nuclear bone scan showing a "hot area" corresponding to the area of lucency. Again this was thought to be metastatic given the increase in radioisotope uptake which is not usual for the lytic bone disease in myeloma. Computerised Tomography scan showed a lesion extending into ulnar styloid process but no cortical destruction. Haematological and blood biochemical tests revealed a haemoglobin of 10 g/dl, white blood cell count of 4.0 × 10^9^/l, platelet count of 164 × 10^9^/l. Her serum globulins were elevated at 68 g/l, calcium was not elevated and the erythrocyte sedimentation rate was elevated at 52 mm/hr. Serum Protein electrophoresis revealed a monoclonal band in γ region with decreased normal immunoglobulins. Bone marrow examination revealed 60 % plasma cells (figure 2). Multiple Myeloma was diagnosed based on the presence of >30% plasma cells in the bone marrow, the monoclonal band and the lytic skeletal lesion. She was treated with melphalan, prednisone and allopurinol which she tolerated well and the pain in the wrist improved. She was advised then by the haematologists to discontinue tamoxifen as she had already had 5 years of treatment.

In December 2001, ten months after stopping the tamoxifen, she was again seen because of an episode of vaginal bleeding which lasted 3 days. The bleeding had been moderate and unprovoked. The history of breast cancer and prolonged tamoxifen use were noted. She had no family history of cancer or of being immunocompromised. and no history of exposure to industrial toxins.

**Figure 3:**
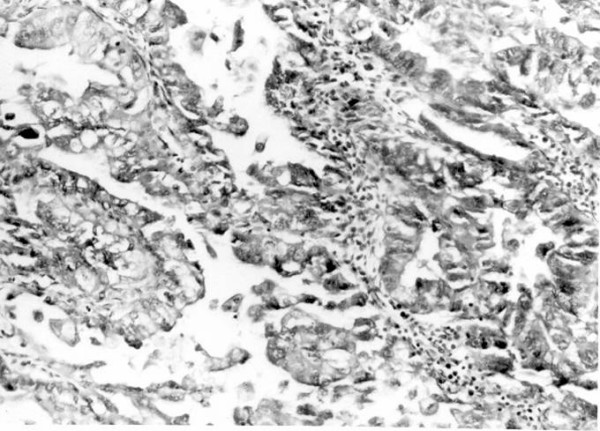


Gynaecological examination was normal except for her uterus of 14 weeks size attributed to uterine fibroids. The cervix appeared normal and a cervical smear was reported as normal. Hysteroscopy was performed which showed a fundal lesion which was removed by suction curettage. The pathology report was that of a poorly differentiated adenocarcinoma (figure 3). She was thus counseled and consented to have a total abdominal hysterectomy. Preoperative investigation revealed Haematological and blood biochemical tests revealed a haemoglobin of 9.6 g/dl, white blood cell count of 5.7 × 10^9^/l, platelet count of 174 × 10^9^/l. Her serum globulins were elevated at 53 g/l, calcium was not elevated, normal blood sugar, normal liver function, normal renal function and a normal chest radiograph. Her serology for HIV and HTLV1 were both non reactive. She had a total abdominal hysterectomy with bilateral salpingo-oophorectomy. The histology from this procedure was similar to that obtained at suction curettage. Staging from the surgery and histology placed her in the category of 1b disease. Unfortunately she died two years later from metastatic disease from this malignancy.

## Conclusion

The main risk factor in this patient appears to have been the long term use of the drug tamoxifen. This is a selective estrogen receptor modulator and a known risk of endometrial cancer and sarcomas [2-4]. Tamoxifen is useful as adjuvant treatment of surgically excised breast cancer. It is usually reserved for oestrogen receptor positive breast cancer patients. With its use, recurrence is decreased by 50%, mortality decreased by 28% [5] and there is a lower incidence of contralateral breast cancer. In one placebo, double blind randomised trial; there was a 49% reduction in breast cancer in high risk women [6]. However since it is not without complication, patients should be informed of the risks which include venous thromboembolism, cataracts and endometrial cancer. The current standard recommendation for use of tamoxifen as adjuvant treatment for breast cancer is 5 years. Use for longer than five years has not been shown to give any added benefit and increases the risk of endometrial cancer [7]. Long term users of tamoxifen also appear to over express the p53 protein on immunohistochemical analysis and this protein is strongly associated with sarcomas and poorly differentiated endometrial carcinomas of the endometrium as was found in this patient who had taken tamoxifen for about seven years [7]. Risk ratio for endometrial cancer is about two and a half to seven times normal in patients being treated with tamoxifen [7]. Non invasive screening procedures such as ultrasonographic endometrial thickness measurement may be beneficial as it has been shown that an endometrial thickness of less than 5 mm is not usually associated with endometrial cancer [8]. However while this has been studied in women with postmenopausal bleeding less is known about it in women on tamoxifen.

The occurrence of the multiple myeloma (MM) appears to have been just a chance event. In this case breast cancer is the most common cancer in Jamaican women Age standardized rate (ASR) 43.2/100,000 (incidence at age 60 173.1/100,000) [9] and multiple myeloma is common in this age group with a reported incidence of 29.3/100,000 at age 60 years (ASR 3.4/100,000) [9]. Endometrial cancer is also common in this age group reported incidence 50.6/100,000 (ASR 9.8/100,000) [9]. Successful treatment of one cancer will result in the patient living long enough for another age related cancer to arise by chance. This phenomenon has been alluded to in a report from Martinique of adult T cell lymphoma occurring by chance with MM, because in that country HTLV1 (associated with ATL) is endemic and MM is common [10].

The occurrence of a bone lesion was at first thought to be a metastatic lesion from the breast however her other studies done confirmed MM which required a different treatment which was successful. A second malignancy should be suspected if the bone lesion is atypical or if the blood studies are not in keeping with breast cancer.

The occurrence of the other problems found in this patient may also be linked to her predisposition to malignancy. Oxidative DNA damage is significantly increased in the trabecular meshwork of glaucoma patients. One study found that Genotypes of glutathione S-transferase isoenzymes were significantly higher in glaucoma patients than in controls. Genotypes of glutathione S-transferase iso-enzyme GSTM1 gene deletion, has been associated with an increased risk of cancer at various sites [11]. Unfortunately the genotype of this patient is unknown.

## Abbreviations

BRCA Breast Cancer

HTLV1 Human T cell Lymphotropic Virus

HPV Human papilloma Virus

HIV Human immunodeficiency Virus

DNA Deoxyribonucleic acid

MM Multiple myeloma

ATL Adult T cell Lymphoma

GSTM1 Glutathione S-transferase iso-enzyme

H&E Haematoxylin and Eosin

## Competing interests

The author(s) declare that they have no competing interests.

## Authors' contributions

H F Gynaecologist involved in care of the patient for the uterine carcinoma and drafted the manuscript. GW, Oncologist who diagnosed multiple myeloma and treated the patient with chemotherapy. EW Pathologist who diagnosed breast cancer. BH. Pathologist who diagnosed uterine carcinoma. DM General surgeon who did mastectomy and treated patient with tamoxifen. All authors read and approved the final manuscript.

## References

[B1] Robison LL Mertens AC Boice JD Breslow NE Donaldson SS Green DM Li FP Meadows AT Mulvihill JJ Neglia JP Nesbit ME Packer RJ Potter JD Sklar CA Smith MA Stovall M Strong LC Yasui Y Zeltzer LK Study design and cohort characteristics of the Childhood Cancer Survivor Study: a multi-institutional collaborative project Med Pediatr Oncol 2002 38 229 39 10.1002/mpo.1316 11920786

[B2] De Myylder X Neven P De Somer M Van Belle Y Vanderick G De Muylder E Endometrial lesions in patients undergoing tamoxifen therapy Int J Gynaecol and Obstet 1991 36 127 130 10.1016/0020-7292(91)90767-Y 1683314

[B3] Evans M Langlois NE Kitchener HC Miller ID Is there a link between long term Tamoxifen and MMT of the uterus Int J Gyne Cancer 1995 5 310 13 10.1046/j.1525-1438.1995.05040310.x 11578496

[B4] Okada DH Rowland JB Petrovic LM Uterine pleomorphic rhabdomyosarcoma in a patient receiving Tamoxifen Gyne Oncol 1999 75 509 13 10.1006/gyno.1999.5604 10600318

[B5] Early breast cancer trialists' collaborative group Tamoxifen for early breast cancer; an overview of the randomized trials Lancet 1998 351 1451 1467 10.1016/S0140-6736(97)11423-4 9605801

[B6] Fisher B Constantino J Wickerman D Redmond C Kavanah M Cronin W Robidoux A Bevers TB Kavanah MT Atkins JN Margolese RG Runowicz CD James JM Ford LG Wolmark N Tamoxifen for prevention of breast cancer: report of the National surgical adjuvant breast and bowel project J Natl Cancer Inst 1998 90 1371 88 10.1093/jnci/90.18.1371 9747868

[B7] Bergman L Beelen M Gallee M Hollema H Benraadt J Leeuwen F The Comprehensive Cancer Centres' ALERT group. Risk and prognosis of endometrial cancer after tamoxifen for breast cancer Lancet 2000 356 881 887 10.1016/S0140-6736(00)02677-5 11036892

[B8] Granberg S Wikland M Karlsson B Norstrom A Friberg LG Endometrial thickness as measured by endo-vaginal Ultrasonography for Identifying Endometrial Abnormality Am Jour of Obstet Gynecol 1991 164 47 52 10.1016/0002-9378(91)90622-x 1986624

[B9] Hanchard B Blake G Wolff C Samuels E Waugh N Simpson D Ramjit C Mitchell K Age specific Incidence of cancer in Kingston and St Andrew, Jamaica 1993–1997 West Ind Med Jour 2001 50 123 129 11677908

[B10] Besson C Gonin C Brebion A Delaunay C Panelatti G Plumelle Y Incidence of Hematological malignancies in Martinique over representation of MM and ATL Leuk 2001 5 828 31 10.1038/sj.leu.2402040 11368445

[B11] Izzotti A Sacca SC Cartiglia C De Flora S Oxidativedeoxyribonucleic acid damage in the eyes of glaucoma patients Am J Med 2003 114 638 46 10.1016/S0002-9343(03)00114-1 12798451

